# The influence of nursing home managers’ leadership on person-centred care and stress of conscience: A cross-sectional study

**DOI:** 10.1186/s12912-021-00718-9

**Published:** 2021-10-15

**Authors:** Annica Backman, Karin Sjögren, Hugo Lövheim, Marie Lindkvist, David Edvardsson

**Affiliations:** 1 Department of Nursing, Umeå University, SE-901 87 Umeå, Sweden; 2 Department of Community Medicine and Rehabilitation, Umeå University, Umeå, Sweden; 3 Epidemiology and Global Health, Umeå University, Umeå, Sweden; 4 School of Nursing and Midwifery, La Trobe University, Melbourne, Australia

**Keywords:** Leadership, Management, Person-centred care, Stress of conscience

## Abstract

**Background:**

Leadership and stress are common concepts in nursing, and this study explores empirically the connection between leadership and stress of conscience in the context of aged care practice. Previous literature has shown that when staff are unable to carry out their ethical liabilities towards the residents, feelings of guilt may occur among staff, which may be an expression of stress of conscience. Although leadership has been described as crucial for staff’s work perceptions of stress as well as for person-centred practices, the influence of nursing home managers’ leadership on stress of conscience among staff and person-centred practices is still not fully explored. This study attempts to address that knowledge gap by exploring the relationship between leadership, person-centred care, and stress of conscience.

**Methods:**

This study was based on a cross-sectional national survey of 2985 staff and their managers in 190 nursing homes throughout Sweden. Descriptive statistics and regression modelling were used to explore associations.

**Results:**

Leadership was associated with a higher degree of person-centred care and less stress of conscience. A higher degree of person-centred care was also associated with less stress of conscience. The results also showed that leadership as well as person-centred care were individually associated with lower levels of stress of conscience when adjusting for potential confounders.

**Conclusion:**

Nursing home managers’ leadership was significantly associated with less staff stress of conscience and more person-centred care. This indicates that a leadership most prominently characterised by coaching and giving feedback, relying on staff and handling conflicts constructively, experimenting with new ideas, and controlling work individually can contribute to less staff stress as well as higher degree of person-centred care provision.

## Background

Previous research has shown that nursing home care services are physically and emotionally challenging with ethical dilemmas and conflicting demands in daily care [1–3]. The prevalence of stress-related illnesses among nursing home care staff has increased during the last decades [4, 5]. On a daily basis, many care situations contain ethically difficult issues and challenges for care staff [6]. Experiences of not being able to fulfil ethical obligations to residents commonly lead to feelings of guilt and stress of conscience [7–9]. Although leadership has been advocated as a key component for staff perceptions of work stress as well as for person-centred care, the impact of nursing home managers’ leadership on levels of stress of conscience are yet to be explored.

In care, nursing home staff balance the ideals and ethical values with the various and sometimes incompatible demands from residents, colleagues, superiors, and organisations [7]. Ethical challenges such as dissonance between residents’ autonomy and a fair and equal resource distribution while still fulfilling organisational demands have been reported as a source to stress in nursing homes [6]. A troubled conscience has been described to occur when direct care staff perceive being unable to do “good” in care, or when they are not able to provide care that corresponds to their ethical values [7]. The concept “Stress of conscience” captures the stress that is generated by a troubled conscience [9, 10]. Lower levels of person-centred care have been associated with higher levels of stress of conscience among nursing home staff [11]. Negative effects of stress of conscience have been well documented with associations with high levels of emotional exhaustion and burnout among staff [12, 13], sleeping problems, and depressive symptoms [14].

Person-centred care has increasingly been promoted as the recommended humanistic model of care that has been adopted by policy-makers, researchers, nursing curriculums, and practitioners due to the ethical underpinnings and emerging evidence of positive effects on residents [15–17] and staff [11, 18]. Person-centred care proposes a humanistic philosophy and ethical values for maintaining the person’s personhood despite illness and integrating the persons wishes and preferences by using personal experiences to individualise both the care and the environment [19]. It seems reasonable to hypothesise that providing person-centred care could reduce perceptions of stress of conscience because providing person-centred care with an emphasis on residents’ wishes and needs would seem to correspond well with common humanistic ethical values and staff ambitions to do good for vulnerable residents. This can in turn be linked to less experiences of stress of conscience. Previous cross-sectional studies have reported that a higher degree of person-centred care is associated with lower levels of stress of conscience [11, 14]. However, a recent multi-centre person-centred intervention study showed no effect on stress of conscience after the intervention. One interpretation by the authors was that the lack of effect was partly due to absent leadership support during the intervention as well as a limited focus on stress of conscience per se [20]. The importance of nursing home managers’ leadership for the implementation of person-centred care has been established by previous research [21–26], as well as the central role of leaders in developing and supporting person-centred care practices in everyday care [27–29]. However, the extent to which leadership is associated with direct care staff experiences of stress of conscience has received limited attention in the literature. Only one study was found that reported a high level of person-centred care and positive leadership as contributing to low stress of conscience among nursing home staff, and that study concluded that leadership support was needed for reducing staff stress of conscience and for enabling personalised care provision [14]. This indicates that leadership may be of significance for both perceptions of stress of conscience and for the extent to which staff are able to provide person-centred care, but existing empirical data are limited, suggesting a need for further research. This study attempts to address this gap in the literature by exploring relationships between leadership, person-centred care, and stress of conscience as perceived by direct care staff.

## Method

### Design and sampling

The present study is based on data from a project with a repeated cross-sectional design, the Swedish National Inventory of Care and Health in Residential Aged Care (SWENIS), carried out within the Umeå Aging and Health Research Programme (U-AGE), [30]. The U-Age SWENIS study uses a repeated cross-sectional design for longitudinal monitoring of health and care in Swedish nursing homes. This study draws solely on SWENIS II data from the follow-up in 2018–2019. The SWENIS II sample consists of data from 43 municipalities (28 baseline and 15 new) contributing with organisational, staff, and resident data from 190 nursing homes.

### Study context

There are approximately 2300 nursing homes in Sweden with approximately 82,000 residents. Nursing homes in Sweden are defined as a specialised form of housing for older people aged 65 years or older who have comprehensive needs for medical care and/or personal care as a result of physical and/or cognitive impairment [31, 32]. The size of the nursing homes in this study ranged between 6 and 116 beds with a mean of 40 beds (SD 18.4) (median 36). Participating units included both general nursing home units (68%) and special care units for people with dementia (32%). Approximately 85% of the nursing homes were run by public providers, while 15% had private providers.

### Data collection

For the purposes of this study, data from two surveys within the SWENIS II were used. The first survey was directed to staff and comprised staff demographic variables (age, gender, native language, educational qualifications, work shift, years of experience in aged care, and years of experience in the current facility). The first survey also asked about staffs’ estimations of leadership, person-centred care, and stress of conscience. The second survey was directed to managers and comprised demographics (age, gender, work experience, and educational qualification) of managers of the included nursing homes (Table 1). The initial step was to obtain consent to conduct the study from the chief executive officer in each municipality. When consent was obtained, the nursing home managers in the invited nursing homes were telephoned and verbal information was given and an e-mail was sent to the nursing home managers with written information about the study. The anonymous surveys were handed out to staff by their nursing home managers. Every completed staff survey was returned in a sealed envelope to safeguard staffs’ confidentiality. The organisational survey was completed by the nursing home managers through a structured telephone interview. One inclusion criterion was that staff needed to have long-term substitution or permanent employment and work daytime or day/evening shifts to be eligible for inclusion. A total of 5803 surveys were distributed in SWENIS II. After removing blank surveys and those that were answered by staff who solely worked night-time, the final sample consisted of 2985 staff (response rate = 52%). The data collection was performed during a 6-month period between November 2018 and April 2019.

**Table 1:** Characteristics of the sample staff *n* = 2985 and managers *n* = 214

**Staff**	**n**^**a**^ **(%)**	**m (SD)**
**Age** (Years)		45.4 (12.3)
**Gender**		
Men	202 (6.8)	
Women	2770 (93.2)	
**Native language**		
Swedish	2313 (82.6)	
Other	487 (17.4)	
**Qualifications**		
Registered nurses	24 (0.8)	
Enrolled nurses	2534 (85.5)	
Nurse’s assistants	296 (10.0)	
No formal qualifications	72 (2.4)	
Other education	37 (1.2)	
**Work shift**		
Day shift	74 (2.5)	
Day and evening	2563 (85.9)	
Day, evening, night shift	304 (10.2)	
Years of experience in aged care		17.2 (11.8)
Years of experience in this facility		9.6 (8.7)
**Managers**	**n**^**b**^**%**	**m (SD)**
**Age** (Years)		50.6 (9.9)
**Gender**		
Men	19 (8.9)	
Women	195 (91.1)	
**Qualifications**		
Human resource specialist	14 (6.5)	
Registered nurses	54 (25.2)	
Enrolled nurses	31 (14.5)	
Social work	89 (41.6)	
Other education	26 (12.1)	
**Leadership education**		
National leadership education 30 ECTS	23 (15.6)	
Other leadership education	124 (84.4)	
Years of experience as a manager		12.7 (9.0)
Years of experience in this facility		4.6 (4.6)

^a^ n does not always add up to 2985 in all variables due to missing items

^b^ n does not always add up to 214 in all variables due to missing items

### Instrumentation

#### Leadership behaviour questionnaire

The Leadership Behaviour Questionnaire was used to investigate leadership and the extent to which direct care staff perceived that their manager expressed a range of behaviours facilitating this [33, 34]. The Leadership Behaviour Questionnaire consists of 24 items formulated as statements about leadership behaviours, where higher scores indicate leadership behaviours that facilitate efficient service delivery [33, 34]. Staff were asked to rate their manager/leader on a six-point scale from 1, completely disagree, to 6, completely agree. A total score can be calculated with a possible range of 24–144 [14]. The Leadership Behaviour Questionnaire has been tested in Swedish aged care contexts in previous research, and the confirmatory factor analysis suggested a unidimensional factor structure with a reliability α = 0.98 [28]. Based on this, the scale has been further explored with a two-parameter item response theory approach to identify the measured leadership characteristics. The most significant behaviours of managers with the highest leadership sum scores were *Experiments with new ideas*, *Controls work closely*, *Relies on his/her subordinates*, and *Coaches and gives direct feedback* followed by *Handles conflicts in a constructive way* [29]. This was interpreted as representing the five most important characteristics of leadership behaviours in nursing homes, were higher scores are favourable. The 24-item questionnaire and its previous psychometric testing is further described in [29, 33, 34]. Cronbach’s α = 0.98 in this sample.

#### Person-centred care assessment tool

The P-CAT is a self-reported assessment scale that measures the extent to which staff perceive the care provided to be person-centred [35, 36]. The P-CAT consists of 13 items formulated as statements about the content of care and aspects of the environment and the organisation. Staff are asked to rate the items on a five-point scale ranging from 1, disagree completely, to 5, agree completely, and a total score is calculated (range 13–65) with higher values indicating a higher degree of person-centred care. The Swedish version of the P-CAT has shown satisfactory estimates of reliability (Cronbach’s α = 0.75) and construct validity in Swedish aged care [36]. Cronbach’s α = 0.77 in this sample.

#### Stress of conscience

The Stress of Conscience Questionnaire assesses stress related to troubled conscience among staff [10]. Stress of conscience arises when staff experience intrapersonal conflicts in relation to a person’s basic values and their views of what is right or wrong and what is good or bad. The questionnaire contains two parts, A and B. Part A comprises nine items and focuses on how often a certain stressful care situation is perceived to occur in the participant’s workplace, with the response alternatives ‘never’ (0), ‘less than once/six months’ (1), ‘more than once/six months’ (2), ‘every month’ (3), ‘every week’ (4), and ‘every day’ (5). Each item in part A is followed by a B question concerning the degree of troubled conscience each specific situation evokes. A continuous variable can be calculated as a measure of stress of conscience by multiplying part A of an item (how often a certain situation is perceived) with part B of the same item (level of troubled conscience the situation evokes). The scores of A and B are multiplied to reflect the total “stress of conscience” for each item. Adding the scores of all items gives a total score with a possible range of between 0 and 225, where higher scores indicate more stress of conscience. The questionnaire is validated for Swedish conditions, and satisfactory psychometric properties have been reported [10]. Cronbach’s α = 0.90 for whole scale (A + B) in this sample, and α = 0.81 for the A part, and α = 0.85 for the B part.

### Analyses

SPSS statistics version 26 was used to analyse the data. Missing items in the questionnaires were imputed with the mean value of the individual for the whole scale. Up to three missing items were replaced in the Leadership Behaviour Questionnaire, (missing < 10.4% of data). For the P-CAT, up to two items were replaced (missing < 7.7% of data) and for Stress of Conscience Questionnaire up to one item was replaced (missing < 7.1% of data) (cf. [37]).

The tolerance coefficient of 0.9 and VIF coefficient of 1.1 were not found to violate the prerequisites for multicollinearity (cf. [38]). No correlation between the scales exceeded *r* = 0.5. The data were checked for potential interaction patterns, and no significant interactions between study variables were discovered. Cronbach’s alpha was used to explore the reliability of all included scales. *P*-values of < 0.05 were regarded as statistically significant, and staff demographic variables (aged, gender, educational qualifications, work shift, and years of experience in aged care and in the current facility) were used for presenting characteristics of the study participants (Table 1) and for controlling for confounders in regression analyses. Managers’ demographics (age, gender, work experience, and educational qualification) and organisational characteristics’ such as operational form, type of setting, and number of beds were used to report characteristics of the managers and the nursing homes and for controlling for confounders in regression analyses.

As a first step, descriptive and comparative statistics were used to explore mean values, standard deviations, and the sample characteristics. As a second step, the correlation between the main study variables was explored by using Pearson’s product-moment correlations. As a third step, a multiple regression analysis was conducted to explore the relationship between leadership, person-centred care, and stress of conscience, with stress of conscience as a dependent variable (adjusted for staff gender, age, education, years of experience in aged care, years of experience in the nursing home, type of facility, operational form, and number of beds per facility). Finally, due to the fact that individuals were correlated in units, e.g. staff rated their perception of practice within the unit, an exchangeable correlation structure was assumed and the parameters were thereby estimated by using Generalised Estimating Equations (GEE) using care unit as the subject variable and stress of conscience as the dependent variable.

## Results

The majority of the participating staff were women (93.2%) with a mean age of 45.4 years (SD ± 12.3). Most participating staff were enrolled nurses (85.5%) with a median work experience within aged care of 17.2 years (SD ± 11.8). Most staff had Swedish as their native language. The majority of the managers were women (91.1%) with a mean age of 50.6 years (SD ± 9.9). They had on average 12.7 years of work experience in aged care (SD ± 9.0) and around 4.6 years (SD ± 4.6) of work experience in their current nursing home. The majority of the managers had a degree in social work (41.6%), followed by registered nursing (25.2%) and enrolled nursing (14.5%) qualifications. About 12.1% of the managers had other education (Table 1).

Leadership and person-centredness of care showed a significant positive correlation (*r* = 0.42, *p* < 0.001). There was also a significant negative correlation between leadership and stress of conscience (*r* = − 0.32, *p* < 0.001). A significant and negative correlation was also found between person-centred care and stress of conscience (*r = −* 0.49, *p* < 0.001). For mean values of leadership, person-centred care, and stress of conscience and their correlations, see Table 2.

**Table 2:** Mean values and correlations of study variables

	M (sd)	*r*	*r*	*r*
		Leadership	SCQ	P-CAT
Leadership	112.1 (22.8)			
SCQ	41.5 (37.0)	−0.32^a^		
P-CAT	50.8 (7.3)	0.42^a^	−0.49^a^	

*SCQ* Stress of Conscience Questionnaire

*P-CAT* Person-centred Care Assessment Tool

^a^ Correlation is significant at the 0.01 level (2-tailed)

In the multiple regression analysis, leadership (β = − 0.22, *p* < 0.001) and person-centred care (st β = −.2.319, *p* < 0.001) were significantly related to a lower level of perceived stress of conscience. The model was adjusted for potential confounders, including staff gender, age, education, years of experience in aged care, years of experience in the nursing home, type of facility, operational form, and number of beds per facility. The multiple regression model explained 28.5% of the variance in stress of conscience (Table 3). The GEE showed similar results, that leadership (β = − 0.224, *p* < 0.001) and person-centred care (β = −.2197, *p* < 0.001) were significantly related to lower level of perceived stress of conscience, even when taking into account that individuals were correlated in units and adjusting for staff gender, age, education, years of experience in aged care, years of experience in the nursing home, type of facility, operational form, and number of beds per facility.

**Table 3:** Multiple linear regression model explaining the variance of stress of conscience

	Linear regression^**a**^			GEE^**a,b**^		
	β	Std. Error	***p***-value	β	Std. Error	***p***-value
Leadership	−0.220	0.032	< 0.001	−0.224	0.0386	< 0.001
Person-centred care	−2.319	0.100	< 0.001	−2.197	0.1171	< 0.001
Dependent variable: Stress of conscience	Adjusted R-Square 28.5%					

^a^ Models adjusted for potential confounders (staff gender, age, education, years of experience in aged care, years of experience in the nursing home, type of facility, operational form, and number of beds per facility)

^b^ *GEE* Generalized Estimating Equations

## Discussion

This study aimed to explore relationships between leadership, person-centred care, and stress of conscience as perceived by direct care staff. The results showed that leadership was associated with higher ratings of person-centred care and lower perceived stress of conscience. Higher ratings of person-centred care were also associated with lower perceived stress of conscience. The results also showed that leadership as well as person-centred care were individually associated with lower levels of stress of conscience in the multiple regression models when adjusting for potential confounders. This indicates that leadership most prominently characterised by coaching and giving feedback, relying on staff, handling conflicts constructively, experimenting with new ideas, and controlling work individually may contribute to less staff stress of conscience, even when adjusted for person-centred care. This has some potential clinical implications for nursing managers who may be in a position to use these leadership behaviours in their own practice to influence person-centred care and to reduce staff stress of conscience. Because there was a positive correlation between leadership and person-centred care, this can also be interpreted as meaning that leadership has an impact on staff stress of conscience partly by positively influencing the provision of person-centred care. This can be interpreted to mean that managers might be able to further promote person-centred care by using the leadership behaviours of trusting staff, delegating responsibility, encouraging thinking along new lines, and discussing and encouraging new ideas. Orrung Wallin et al. [14] also using the same leadership scale showed that these leadership dimensions are important for staff’s perception of stress of conscience, and this current study supports their findings.

Direct care staff in nursing homes might be confronted with ethical dilemmas in their daily work and are sometimes left alone in these difficult choices and decision-making, thus potentially bringing a burdensome and mentally demanding work situation [39]. Although a conscience can be perceived as an asset, a warning signal that makes you act in accordance with your moral values [40], feelings of powerlessness and inadequacy may occur when the conscience is constantly challenged when striving to be and do “good enough” in your work [41]. Previous research has shown that the perception of stress of conscience can arise if and when limited support and leadership are perceived from managers, and this is why leadership seems to play an important role for perceived stress of conscience [7]. It has been stated that staff need opportunities to discuss conflicting feelings to be able to cope with the consequences that follow a troubled conscience [7], and by sharing experiences with others an agreement in the work group on what can be seen as reasonable goals and requirements in daily care can be achieved [42, 43]. Seen in relation to this study’s findings, where nursing home managers’ leadership significantly impacts levels of stress of conscience among staff, an important implication for managers seems to be to facilitate ethical discussions so that staff can achieve a balance between conflicting demands and can discuss what can be considered as realistic measures in everyday care. Previous research has shown that staff who have to deaden their conscience to be able to work and simultaneously perceive low support are at risk for burnout [43], giving further support for the centrality of leadership and support. A previous study has also pointed out that a manager needs to identify and handle organisational factors that are ethically relevant, especially when these contradict the staff’s value standards [44]. This indicates that nursing home managers need to reflect upon the organisational context within nursing homes, and recognize when there is a mismatch between care needed and care provided, and assist managing these dilemmas, as this has significance on staff perception of stress of conscience. One important implication for policy makers and stakeholders is to consider the range and complexity of conflicting ethical principles that staff encounter in their daily work, and provide support and resources for regular team reflections’, so that diverse perspectives can brought up for open discussion as a strategy to mitigate stress of conscience among staff.

The results in the current study also showed that a higher degree of person-centred care was associated with lower levels of stress of conscience. This is consistent with previous research [11, 45] suggesting that working in a person-centred manner may mean providing care in line with one’s intrinsic values and one’s own perceptions of “good care”. This is further supported by intervention research by Edvardsson et al. [46] where “doing good” for residents (i.e. providing the care and activities as wanted/whished) reduced perceptions of stress of conscience among staff. This in turn has implications on staff intentions to leave the field because lack of support, appreciation, and resources hinders staff in providing care as they wish to, which contributes to staff turnover in nursing homes [47]. The findings of this study also revealed a significant association between leadership and person-centred care, highlighting the importance of nursing home managers’ leadership because this can be interpreted as leadership having a direct impact on staff stress of conscience, but also an indirect impact through influencing the development of person-centred care provision. This implies that nursing home managers have an important leadership role in inspiring and making it possible for staff to be able to provide care that is based on residents’ needs and preferences because “doing good” not only enables high-quality care, but also soothes and relieves the conscience among staff. Previous research has suggested that the Stress of Conscience Questionnaire can be a significantly helpful tool for clinical managers in this process [48]. This study is novel and adds some empirical evidence for how leadership is associated with staff stress of conscience and person-centred care, something which has only sparsely been empirically investigated previously. This study provides insights that a leadership most prominently characterised by behaviours such as *experimenting with new ideas*, *controlling work closely*, *relying on his/her subordinates*, *coaching and giving direct feedback*, and *handling conflicts in a constructive way* is positively associated with less staff stress of conscience as well as with increased person-centred care. In terms of practical implications, this suggests that managers can employ these concrete behaviours as a means to improve the person-centredness of care and to mitigate the workforce risks and consequences of staff stress of conscience.

### Limitations

This study is based on self-rated cross-sectional data from staff, which means that inferences about causality are not possible. However, the direction of analyses was based on previous theoretical hypotheses lending support for the analyses that were used. Still, subsequent longitudinal and interventional studies are recommended. This study draws on data from the Swedish aged care context, and it remains unknown whether or not the results would differ if other participants in other nursing contexts responded to the survey. However, this study is based on extensive cross-sectional randomised follow-up data from a national representative sample of staff in Swedish nursing homes, which suggests that the results might be applicable across similar contexts, but we leave it to the reader to make this judgement of transferability. The regression models explained 28% of the variance in stress of conscience in a linear regression model when including the demographic variables of the organisation. This suggests that there are other unknown factors that can contribute to explaining stress of conscience among staff in nursing home care. This suggests that further research is needed to explore additional factors, for example, staff workload, preparedness to handle and cope with difficult ethical situations, etc. Further international comparative and interventional studies would be valuable in confirming or rejecting these findings and in exploring the trajectories and interventions that might promote leadership in nursing home care and the associated stress of conscience and person-centred care.

## Conclusion

This study provides important measures of leadership in nursing home care and its significance for stress of conscience and degree of person-centred care. The results show that nursing home managers’ leadership is significantly correlated to less staff stress of conscience and more person-centred care provision. This indicates that a leadership most prominently characterised by coaching and giving feedback, relying on staff, handling conflicts constructively, experimenting with new ideas, and controlling work individually can contribute to less staff stress as well as increased person-centred care provision. This can be interpreted to mean that nursing home managers have an important leadership role in inspiring and enabling staff to provide person-centred care that is based on residents’ needs and preferences and that this may, in addition to improving ethical, humanistic, and high quality care as shown in other studies, also lessen the stress of conscience in direct care staff.

## Data Availability

The datasets analysed in the current study available from corresponding author on reasonable request.

## Abbreviations

OECD: Organisation for Economic Co-operation and Development
NBHW: National Board of Health and Welfare
P-CAT: The Person-centred Care Assessment Tool
SCQ: Stress of conscience
